# Towards ethical good practice in cash transfer trials and their evaluation

**DOI:** 10.12688/openreseurope.14258.2

**Published:** 2022-03-21

**Authors:** Neil Howard

**Affiliations:** 1 Department of Social & Policy Sciences, University of Bath, Bath, UK

**Keywords:** Cash transfers, trials, pilots, evaluation, ethics, RCTs

## Abstract

Over the past 20 years, cash transfers have become increasingly widespread within international development and global social policy. Often, their roll out is preceded by a trial or pilot phase aiming to check feasibility and effectiveness. These pilots can involve thousands of people. However, there is limited discussion within the literature (and even less in practice) of how and whether cash transfer trials and the research that they involve can respect ethical standards. This paper represents an initial step towards filling that gap. It does so by reviewing the latest literature pertaining to the ethics of cash transfers and social experimentation. It concludes by advancing a series of proposals that could support cash transfer trials to take place with greater respect for research ethics norms and in the best interests of participants. The paper’s findings have relevance for policymakers and development practitioners working with cash transfers and also for the smaller cognate world of Unconditional Basic Income (UBI) piloting.

## Plain language summary

Although cash transfers are now widely used within development and social policy, there is still limited discussion over how (and indeed whether) cash transfer trials and research on them can respect ethical standards. This paper assesses the latest relevant literature and advances a series of proposals that may support cash transfer trials to take place ethically and with respect for the best interests of participants. The paper may be of interest to scholars and practitioners engaged in cash transfer or basic income piloting or in experimental/trial-based research more broadly.

## Introduction

Since their emergence in the 1990s, cash transfers have spread exponentially throughout the fields of social and development policy, forming a key part of social protection strategies worldwide ( Bastagli *et al.,* 2016). Defined as “direct, regular and predictable non-contributory payments that raise and smooth incomes with the objective of reducing poverty and vulnerability” ( DFID, 2011: 2), the success of the cash transfer ‘travelling model’ ( Olivier de Sardan & Piccoli, 2018a) has been so great that cash transfers have become “the main form of intervention channelled in the direction of the most vulnerable families in low- and middle-income countries (LMICs)” (ibid.: 1). One recent study estimated that, pre-COVID-19, as many as 130 countries had cash transfer programmes, with another calculating their share of total worldwide humanitarian aid to exceed 10 percent ( CALP, 2018; also see Bruers, 2019; Davis *et al.,* 2016: iv). In the context of COVID-19, each of these figures has increased significantly ( Gentilini *et al.,* 2020).

The spread of the cash transfer model is in large part attributable to how efficient and effective cash transfers have been at achieving policy goals. Pioneering programmes in Mexico and Brazil, for example, aimed at increasing school enrolment amongst poor communities and succeeded unambiguously ( Akresh *et al.,* 2013). Following this, newer programmes began targeting transfers at different constituencies and to different ends: to the extreme poor to reduce their poverty; to the elderly to reduce their dependency; or to expectant mothers to improve their calorie intake. Research on programmes across all of these domains suggests that transfers have consistently been successful and that their potential for expansion to other domains is high ( Bastagli *et al.,* 2016; DFID, 2011: ii).

In their development phase, many cash transfer programmes begin with a phase of experimental research ^1^ – as trials or pilots which are evaluated and if successful scaled. Typically, the randomised control trial (RCT) is seen as the ‘gold standard’ in trialling and evaluation ( Bédécarrats *et al.,* 2020), since the discourse surrounding RCTs suggests that they can attribute causality in ways that no other method can (e.g., Banerjee & Duflo, 2011) ^2^. RCTs function by selecting individuals who are putatively identical according to specific criteria and then randomly assigning them to treatment and control groups. The treatment – in this case, cash transfers – is administered before statistical tools are used to measure what changed and to what extent this was caused by the treatment.

Although the literatures on cash transfers and on experimental methods (in particular RCTs) are by now ubiquitous, writing that focusses specifically on the ethics of either is still relatively limited (at least outside of the Medical Sciences, which possesses a rich literature on medical RCTs). The Cash Learning Partnership (CALP) ( https://www.calpnetwork.org/), for example, is a global collaboration between humanitarian actors that collectively deliver the vast majority of cash and voucher assistance in emergency contexts worldwide. It brings together governments, the United Nations (UN), and civil society actors, and its website is the largest grey literature repository anywhere related to cash assistance and cash transfers. Tellingly, of the more than 1,200 documents it hosts, only a handful specifically address ethics. This is paralleled in both the development evaluation literature ( Barnett & Camfield, 2016; Groves Williams, 2016) and the smaller, related literature on UBI piloting (e.g. Widerquist, 2018). It is further paralleled in the wider academic literature on experimental social science ( Barrett & Carter, 2010: 519), although this latter has begun to take ethics more seriously, with ethics-related contributions (particularly in relation to RCTs) growing at a rapid rate (see Abramowicz & Szarfarz, 2020; Deaton, 2020; Hoffman, 2020; Kaplan *et al.,* 2020, for recent contributions). It is within this emerging body of work that this paper situates itself.

Methodologically, two approaches were used for gathering the literature examined by this review beyond consulting the CALP database. First, I conducted a literature search using Google Scholar. Google Scholar was chosen because its indexing includes various sources of literature that go beyond traditional academic journals. Various combinations of search terms were used: i) ‘ethics’ or ‘ethical’ and ‘cash transfers’ (a total of eight results); ii) ‘ethics’ and ‘ethical’ with ‘cash transfer pilot’ and ‘cash transfer trial’ (no results); iii) ‘ethics’ and ‘ethical’ with ‘social experiment’ (nine results); ‘ethics’ and ‘ethical’ with ‘experimental research’ (>40 results); ‘ethics’ and ‘ethical’ with ‘pilot’ and ‘trial’ (>300 results). Exclusion criteria included: publications not in the English language; publications that were inaccessible (>10); publications from Medical and Life Sciences (often related to animal experimentation, >100); publications focussed on the teaching of ethics (>50) and on law (<50). Exclusion was operationalised by reading the abstracts for each publication. A final list of 15 eligible pieces was generated and examined. The second approach used to gather literature was to consult specialists in research ethics and in cash transfer research (five), to obtain reading recommendations from them, and to snowball relevant literature on the basis of subsequent reading. These specialists were either known to the author or recommended to him. This led to a significant body of work on the ethics of cash transfers more broadly (>20). The results of this process are cited throughout the paper and included in the bibliography.

Concretely, the paper aims to unpick certain of the tangled ethical knots inherent to cash transfer piloting, which relates necessarily to cash transfer programming. What are the challenges experimental researchers will face in this field? And how might those be overcome? In addressing these questions, the paper draws on insights from anthropology, development studies, economics, medical research, and applied philosophy.

## Ethical challenges

Thinking through the ethical challenges involved in trialling and evaluating a cash transfer pilot requires two key steps. First, assessing the ethical issues relating to cash transfers (and social protection/development) more broadly. Second, examining the issues related specifically to experimental research endeavours such as trials. This section of the paper does both.

### Ethical challenges related to cash transfers

The literature on the ethical questions raised by cash transfer programming identifies three primary issues. These are: i) conditionality, ii) targeting and associated practices of exclusion/inclusion, and iii) sustainability and exit. Each of these matters because under certain circumstances they may lead to harm.

We begin with conditionality. For most of their short history, the preferred design of cash transfer interventions has been conditional, since a common assumption among policymakers has been that, without strict conditionality, programmes will fail to achieve their stated goals (see Dammert *et al*., 2017 for a good overview). Guy Standing is perhaps the most celebrated opponent of this position, arguing that conditions are both unnecessary and unethical:

“By definition, conditions are paternalistic, patronising and contrary to human rights and freedom. They are costly to apply, inefficient and inequitable, and may be counterproductive and create barriers of suspicion and resentment among recipients. They turn policy implementers into interferers, benevolent or otherwise. They also raise moral dilemmas. Suppose an impoverished mother is told that she can receive the payment only if her children go to school every day. If she cannot force her 12-year-old son to go, will the policy-maker take away the money, leaving the woman and son in dire poverty?” ( Standing, 2014: 122).

A wide variety of commentators concur, arguing that conditions represent a top-down exercise of power by the privileged over the vulnerable; fail to respect individual autonomy; undervalue contextual knowledge; and often cause harm through humiliation and increased stigmatisation ( Aste *et al.,* 2018; Balen, 2018; Davala *et al.,* 2015; Piccoli & Gillespie, 2018; Nagels, 2018). On this latter point, there is abundant empirical evidence. The collection of papers in Olivier de Sardan and Piccoli’s recent anthropological study of cash transfer programmes, for instance, shows clearly how often those who police conditionality do so abusively and with many negative psychological effects on recipient populations ( Nagels, 2018; Piccoli & Gillespie, 2018). ^3^ These impacts often carry into the future, as Balen and Fotta show in their recent overview of conditionality in Latin America (2021), which shows how cash transfer governmentality can re-configure social relations over time.

The second key issue here is the use of targeting and associated practices of exclusion/inclusion in cash transfer programming. Every existent cash transfer programme targets in some way, since resources (and political will) are lacking for universal programming. This necessarily means drawing a line between who receives and who does not, and sometimes between who is ‘deserving’ and who is not ( Krubiner & Merritt, 2017). Such line-drawing inevitably creates winners and losers, with important impacts on recipient and non-recipient well-being. For example, in their study of a long-term cash transfer trial in Kenya (in fact, a UBI trial), Haushofer *et al.* (2015: 3) found that, as a result of exclusion, the well-being of non-recipients declined by four times as much as the corresponding increase in well-being among recipients. Similarly, in their South African study, MacPhail *et al.* (2013: 2305–6) found both dissatisfaction among those excluded from the programme and an increase in bad feeling between the included and excluded. Anthropological researchers have begun to delve into these findings in greater depth, finding unsurprisingly that people perceive targeting to be ‘unfair’ and unreflective of local realities and inequalities. This is especially the case when targeting takes place within communities and without full buy-in as to the lines dividing the included and excluded ( Olivier de Sardan & Piccoli, 2018a). In the words of Olivier de Sardan and Piccoli:

“In communities that are characterised as being generally poor, targeting creates an externally imposed threshold effect between beneficiaries and nonbeneficiaries, and, in many cases, this division does not make sense to the populations and appears arbitrary or illegitimate from their perspective.” ( Olivier de Sardan & Piccoli, 2018a: 8).

The third ethical concern here relates to sustainability and exit. Development agencies have long been criticised for short-termism and carelessness when it comes to managing the end of their interventions ( Gardner *et al.*, 2005). The same applies to cash transfer programmes, since some agencies (though by no means all) fail to prepare recipients for the end of their support, in turn jeopardising the sustainability of any gains made. Recipients may, for example, adjust their behaviour in the expectation that support will be ongoing and then struggle to adapt when they learn that it is not ( Hayman *et al.,* 2016; Levinger & Macleod, 2002). Evidently this may cause harm.

### Ethical challenges related to experimental research

We now turn to the ethical challenges relating to experimental research in the social sciences such as cash transfer trials. One of the major contributions to thinking around this issue is Stéphane Baele’s seminal 2013 paper, ‘ *The Ethics of New Development Economics: Is the Experimental Approach to Development Economics Morally Wrong?* In this paper, Baele surveyed the literature on what he calls ‘the experimental approach in development economics’ (by which he primarily meant RCTs) and identified six major, un-addressed ethical problems that appear to plague the field. These are:

1.   The ‘hazardous calculus problem’, or the problem of negative unintended (or even worse, intended) consequences.

2.   The ‘randomisation problem’, which involves treating equal people unequally as a result of randomising across treatment and control groups.

3.   The ‘consent problem’, which relates to the fact that many trials fail to respect individual autonomy by failing to seek informed consent from participants.

4.   The ‘instrumentalisation problem’, which follows Kant’s interdiction against treating people as means not ends and follows from the absence of informed consent.

5.   The ‘accountability problem’, which relates to the responsibility that researchers have towards participants when their experiments have damaging consequences – which often they have been shown to have had.

6.   The ‘foreign intervention problem’, which concerns foreign actors intervening in the affairs of countries of the Global South, at times with a political agenda and at others simply as (neo-colonial) researchers (ibid. 10–30).

Similarly, in their paper, ‘ *The Power and Pitfalls of Experiments in Development Economics: Some Non-random Reflections’* (2010), Christopher Barrett and Michael Carter identify the following four ‘ethical dilemmas’ as widespread and often un-addressed within the field of experimental social science research:

1.   The violation of the ‘do no harm’ principle, which they view as “perhaps the most fundamental ethical obligation of all researchers” ( Barrett & Carter, 2010: 519).

2.   The suspension of informed consent.

3.   ‘The blindness problem’, which relates to randomisation and the fact that people in a control group often experience distress as a result of knowingly missing out on a potentially beneficial treatment.

4.   The targeting problem, which relates to “the unfairness and wastefulness implied by strict randomisation” in a context of scarce resources ( Barrett & Carter, 2010: 521), meaning that people who do not need the treatment nevertheless receive it while those in need do not.

Other commentators echo these concerns and have begun to expand upon them. World Bank researchers Martin Ravallion and Berk Özler argue that experimental trials sometimes violate the ‘do no harm’ principle, including through inciting problematic behaviour among participants ( Özler, 2014; Ravallion, 2014). ^4^ While scholars such as McKenzie (2013), MacPhail *et al.* (2013), and Haushofer *et al.* (2015) all caution against the manifold moral challenges inherent to the process of randomisation.

From this literature, the following list of overarching, interrelated issues can be distilled as of relevance to the ethics of trial-based research around cash transfers. Each will be discussed in turn:

•   Negative consequences that do harm to participants (intended or unintended).

•   The side-effects of randomisation.

•   The instrumentalisation of participants.

•   Informed consent.

•   Researcher accountability.

•   The potential coloniality of foreign intervention.

### Negative consequences that do harm

The ‘do no harm’ principle is seen as foundational by research handbooks of all stripes and by all ethical review boards. In his summary for the European Commission, for example, ethicist Ron Iphofen describes ‘not doing harm’ as one of “the basic ethical principles to be maintained in all research” ( Iphofen, 2011: 1). Doing harm may be intentional or unintentional. Intentional harm refers to harm that is an intrinsic part of the experiment itself and most critics argue that this can only be permissible under strict conditions, namely “negligible consequences [for participants], unambiguous scientific need for the study and its experimental design, and particular importance of the results” ( Baele, 2013: 24). In Barrett and Carter’s words, “Standard human subjects rules require: (1) that any predictable harm be decisively outweighed by social gains; (2) that subjects be fully informed of the risks; and (3) that compensation be paid to cover any damages incurred” (2010: 520). The example of an injection may be instructive. Injections can be painful and are often undesirable, but trials using injections can be acceptable if participants are informed and compensated and if the injection and the research of which it is part are truly scientifically necessary ( Iphofen, 2011: 14).

Unintentional harm is more complicated and the risks of it can be mitigated, even if never fully. Concretely, what mitigation means will vary in any given context and according to the nature of the research in question, but it always involves reflection and action to protect participants, researchers, institutions, and other stakeholders. The kinds of questions that may be asked when seeking to avoid harm include: Who does this research benefit and how? What are the potential risks of the research and to whom? Could harm arise, of a personal, psychological, inter-personal, spiritual or economic nature? Are we, as researchers, acting in integrity and with care, including for ourselves and our colleagues? ( Iphofen, 2011: 24-30; Kaplan *et al.,* 2020). What other ways can we think of to achieve our scientific and social objectives without increasing the risk of harm? Sadly, the literature on experimental social science and particularly RCTs is replete with examples of scholars failing to ask these questions and consequently causing harm. MacPhail *et al.,* for instance, discuss the chilling example of an RCT generating conflict among South African youth ( MacPhail *et al.,* 2013: 2306), while Baele (2013) and Sarin (2019) include a variety of similarly concerning stories.

### The side-effects of randomisation

The overwhelming majority of the emerging literature on the ethics of experimental social science concerns randomisation and its negative, harmful side-effects. To recap, randomisation is the practice of randomly assigning individuals to treatment and control groups in order to facilitate the use of statistical methods for evaluating the effect of the treatment under investigation. Developed and widely deployed in the medical sciences over the past 25 – 30 years, RCTs have become increasingly important for economists in the social sciences over the last 15. But randomisation has several problematic side-effects, and many argue that it is inherently indefensible in certain circumstances.

As Baele (2013) says, the core issue with randomisation is that it treats equal people unequally. From a deontological perspective, this is unacceptable – if two households are equally poor then it is hard to justify giving money only to one of them. Moreover, in practice, there is emerging evidence that treating equal people unequally as a requirement of randomisation can generate resentment, reductions in well-being, and even conflict – unacceptable therefore also from a consequentialist perspective. The examples above from Kenya and South Africa attest to this ( Haushofer *et al.,* 2015; MacPhail *et al.,* 2013). Both were RCTs and in each case recipients were included or excluded randomly. Yet in each case this division was perceived as unfair by some in the control groups; it therefore reduced non-recipient wellbeing and generated conflict. Such a finding merits considerable further exploration, for it challenges the common experimental assumption that being in a control group will carry no costs, since theoretically participants are left no worse off than they would otherwise have been. By contrast, these papers suggest that the ill-being generated by being in proximity to (but not a beneficiary of) good fortune may be greater than the ‘ordinary’ experience of being poor in the face of inequality. Under such circumstances, RCTs (and other forms of randomisation) could be argued to violate the do no harm principle by their very nature. 

### Instrumentalisation of participants

Related to randomisation is the issue of instrumentalisation of research participants. According to Baele, “All [RCT] case-studies manipulate people in order to reveal a scientific result which might be useful to policy-makers willing to reduce poverty; in this, one could argue that the method indeed instrumentalises individuals” ( Baele, 2013: 25–26). Following Kant’s famous argument, Baele considers it wrong to treat people as means rather than ends; this implies that if the subject matter of a study has nothing to do with its participants’ lives and the study offers them no benefit then it will be morally unacceptable because their inclusion is wholly instrumental. Naturally, many experimental researchers push back against this by claiming that even participants in control groups derive benefit and are concerned by their study because the study seeks “to fight against a clearly identified social problem experienced by the participants themselves” ( Miguel & Kremer, 2001 in Baele, 2013: 26).

This discussion points towards the critical ethics question of reciprocity or benefit sharing. It is a widely accepted tenet of ethics protocols that people must derive some benefit from participating in a research project – in the words of Seymour-Smith, researchers must try to “perform some useful or valued service in return for the collaboration require[d]” from participants (Seymour-Smith, 2007: 9 cited in Robben & Sluka, 2007). Yet too often this fails to happen. Participants enrolled in control groups often receive nothing in return for their participation, even when they learn that the target group did ( Baele, 2013; Humphreys, 2015).

### Informed consent

Many of the above problems come back to the absence of informed consent in experimental projects. Remarkably, despite its centrality to ethical guidelines, the requirement to obtain informed consent is very often ignored even in high-profile experimental social science research ( Hoffman, 2020). As Barrett and Carter explain:

“To avoid the various endogenous behavioural responses that call into question even the internal validity of experimental results (due to Hawthorne effects and the like), many prominent studies randomise treatments in group cluster designs such that individuals are unaware that they are (or are not) part of an experiment. The randomised roll-out of Progresa in Mexico is a well-known example…. Even when the randomisation is public and transparent, cluster randomisation maintains the exogeneity of the intervention, but at the ethically-questionable cost of sacrificing the well-accepted right of each individual participant to informed consent, as well as the corresponding obligation of the researcher to secure such consent.” ( Barrett & Carter, 2010: 520).

The basic methodological issue is that it becomes more difficult to attribute causality to the treatment under investigation when participants know that they are part of an experiment and either receiving the treatment or not. Their ignorance is thus “meant to prevent changes in the participants’ behaviours that could threaten the scientific outcome” ( Baele, 2013: 23).

Yet of course this poses ethical problems from both deontological and consequentialist perspectives. Deontologists argue *a priori* that lying is wrong, not least because doing so involves breaking the categorical imperative by treating people as means and not ends. For consequentialists, the issue is more about what is gained from the deception (and, implicitly, coercion, since the abrogation of consent can be read as a form of coercion). Following Bonetti, they view deception as permissible only “when a) its consequences are negligible, b) the scientific enquiry unambiguously requires it, and c) the probable discovery is particularly important” ( Bonetti, 1998: 390). Yet, as researchers from Ravallion (2014) to Hoffman (2020) observe, these criteria are far from always observed in experimental social science research. Plenty of it fails to offer anything like a meaningful scientific discovery ( Baele, 2013: 13), while, as Hoffman observes, abrogating consent de-humanises participants and increases the risks of unintentional harms ( Hoffman, 2020: 2).

### Researcher accountability

The above all points to the question of accountability. In one of the earliest papers to reflect on the question, Humphreys and Weinstein ask, “to what extent are researchers responsible for outcomes that result from manipulations implemented by third parties?” as part of their research ( Humphreys & Weinstein, 2009: 375). Put more broadly, Baele asks: “are researchers accountable for the harmful effects of their RCTs?” ( Baele (2013: 27). In the ethical guidelines he produced for the European Commission, Iphofen notes that “clarifying lines of accountability” is an essential part of ethical review, making clear “who takes decisions, on what grounds and who is responsible for errors and misjudgements” ( Iphofen, 2011: 12). This is indeed well established in the medical sciences where, as Angell has observed, “investigators are responsible for all subjects enrolled in a trial, not just some of them, and the goals of the research are always secondary to the well-being of the participants” ( Angell, 1997: 847). Here legal liability accompanies and enforces moral responsibility, with the consequence that gross malpractice is unlikely to go unpunished.

However, within the experimental social sciences this is less often the case. There are myriad examples within the literature of researchers designing experiments that harm participants. These will presumably have escaped ethical review by lead researchers’ home institutions, possibly because ethical guidelines on experimental methods in the social sciences are still not as widespread as needed. What is required is rigorous risk assessment, critical evaluation, meaningful local partnership, clear lines of responsibility, and plans for compensation in cases of harm ( Baele, 2013: 27–8).

### The potential coloniality of foreign intervention

The final issue raised by this review of the literature is that of coloniality. In her seminal work, ‘ *Decolonizing Methodologies: Research and Indigenous Peoples’* (1999), Linda Tuhiwai Smith argues that “the word itself, ‘research’, is probably one of the dirtiest words in the indigenous world's vocabulary” ( Tuhiwai Smith, 1999: 1). This is both because it underpinned “the worst excesses of colonialism” and because still today it is often used to subordinate and exploit subaltern populations (1999: 1; see also Zavala, 2013). This raises the fundamental questions of who research is designed to benefit, who it may harm in the process, and how these things map onto existing global inequalities.

In her recent contribution to thinking in this vein, Nina Hoffman goes as far as to call for a “moratorium…on experiments in former colonies” ( Hoffman, 2020: 1). Drawing on a systematic review of all RCTs published between 2009 and 2014 in ‘top economics journals’, she found that only 46% discuss whether participants were aware that a study was being conducted. Shockingly, “participant awareness is discussed in 65% of experiments conducted in Europe and the United States, compared with 34% of experiments conducted in Africa, Asia and Latin America…[which] suggests a troubling difference in ethical standards” (ibid. 1). Indeed, Hoffman suggests that this difference is significant both because it implies a racialized coding of standard application and an absence of informed consent. In turn, this suggests that many studies, especially in the Global South, run the risk of both dehumanizing participants and increasing the likelihood of negative unintended consequences (ibid. 2).

Beyond this, there is ample literature suggesting that international research collaborations between the Global North and Global South, of which RCTs and other experimental studies are prime examples, may i) cause significant harm, and ii) entrench existing power relations. On the latter point, it is worth noting with Hoffman that “of the [reviewed] experiments conducted in former colonies, 84% of lead authors were at institutions in the United States or Western Europe”, while “no first authors were located in Africa or Latin America” ( Hoffman, 2020: 2). This strongly suggests that experimental research has the tendency to reproduce hierarchies of power in systems of knowledge-production, which themselves echo the troubling and often painful hierarchies so associated with research in the colonial past (2020: 2). On the former point – the causing of harm – there are myriad ways in which this may take place. Most significant for this discussion, however, is the fact that it matters who interprets ‘what’ and ‘how’, since inaccurate interpretations and subsequent representations can lead to negative consequences for participants, including in the form of disciplinary policy interventions (e.g., Howard & Okyere, 2019; O’Connell Davidson, 2015). Research and ‘knowledge’ production are never neutral, since they take place in conditions of extreme inequality ^5^, and unless this is actively mitigated for there is a risk that ill-informed outsiders may unintentionally cement or even exacerbate it.

## Responding to ethical challenges

Having discussed the ethical challenges identified by the literature in the previous section, the following section presents thinking around how these might be managed in the conduct of ethical experimental research around cash transfers. It is organised following the same structure as in the previous section, beginning with responses related to cash transfer design and subsequently addressing responses specific to experimental design.

### Responses related to cash transfer intervention design

As discussed above, the central ethical issues raised by cash transfer programming include i) conditionality, ii) targeting and associated practices of exclusion/inclusion, and iii) sustainability and exit. We begin with conditionality.

Much of the literature on this suggests that conditions should be done away with entirely, with programmes instead respecting recipients’ autonomy to make free choices over how to use their resources. Guy Standing argues that conditions fail the following two key principles that he believes should be used to evaluate whether a policy is socially just: 1) the Paternalism Test principle, and 2) the Rights-not-Charity principle. According to the Paternalism Test principle, “it is socially unjust to impose controls or directives on some groups that are not imposed on the “most-free” groups”. With the Rights-not-Charity principle, “a policy that extends the discretionary power of bureaucrats or other intermediaries while limiting the rights of recipients is socially unjust” ( Standing, 2014: 113). Beyond injustice, many also argue that conditions are simply ineffective, both because people often ignore them and because recipients typically have greater situated knowledge as to their real needs than programme designers. For thinking in this vein, conditionality of any kind is unjust and undesirable, to be rejected in favour of an unconditional approach that respects recipient autonomy and thus also dignity ( Davala *et al.,* 2015).

Similar anti-restriction arguments surround targeting and exclusion/inclusion. Although well-intentioned – in that it typically aims to maximise beneficial use of limited resources by reaching those most in need – targeting has many critics because it involves creating artificial divisions between similar people and often fosters resentment and conflict. It also typically fails, generating many type one and type two errors (i.e., excluding those who should be included and including those who should be excluded, respectively [ Standing, 2014: 121]) and is frequently subject to abuse ( Olivier de Sardan & Hamani, 2018). Moreover, by definition, targeting involves the imposition of external benchmarks of deservingness on beneficiaries, which in turn reinforces hierarchical, neo-colonial relations of power between them and their donors ( Olivier de Sardan & Piccoli, 2018). To mitigate these issues, one strand of literature argues that we should develop better, more accurate, and more benevolent targeting tools, such as participatory wealth mapping (e.g., Wood & Marsden, 2018) or action research approaches that are guided by the intention to include the full range of perspectives ^6^. Another suggests that targeting should be done away with altogether. This is the position of those who call for UBI.

What of sustainability and exit? The literature on both is clear. Although an obvious case can be made that desirable social policies should be permanent rather than temporary, the positive effects of even time-bound interventions is well established. With cash transfer interventions in particular, we know that these can be long-lasting and sustainable, especially if accompanied by appropriate non-financial support such as coaching or connection to state services ( Davala *et al.,* 2017; Handa *et al.,* 2016; Raza *et al.,* 2012). Crucially, that support must also prepare people for the end of the intervention. First, by ensuring that they fully understand and consent to a programme that is time-bound and by reminding them of the time-bound nature of the programme as it is ongoing, lest there be any surprises. Second, by making sure that all participants have solid practicable individual or household exit plans which can smooth the transition.

How might a cash transfer trial apply these varied insights? One option would be to adopt an unconditional approach to the delivery of cash transfers and to sidestep the targeting problem by distributing transfers universally within selected participant communities. This could further involve selecting participant communities that are discrete, clearly delineated entities of a particular size and socio-economic level. Although such an approach would still involve targeting in the sense that not all communities would receive transfers, it should enable a project to avoid many of the issues documented above in relation to within-community targeting.

With regards to sustainability and exit, any given trial team could include established local civil society actors familiar with participant communities. These would follow good practice guidelines around delivery and exit and have experience in the field ( Gardner *et al.*, 2005; Skovdal *et al.,* 2012). Good practice would include commitments to full transparency with participants at every stage of the project, informed consent, the building of individualised exit and sustainability plans, and putting in place appropriate counselling if needed. In addition, a trial could include a ‘plus’ element involving community workers whose task it would be to collaborate over the life of the trial with community members in a) making the most of the cash received, b) developing non-cash related change plans and resilience, and c) planning for the end of the intervention. A small handful of projects are already developing such an approach (e.g. https://www.work-free.net/).

### Responses related to experimental research design

The rest of the present section will delve into the design of the research around a cash transfer trial, following the list of points outlined in the section above.

### Negative consequences that do harm

The literature is clear that the obvious way to avoid intentionally harming research participants is to design a project that does not do so. Simply put, if a project knowingly harms people or incites damaging behaviour among participants then there is a strong argument that it should not be given ethical clearance to proceed. Following the standard human subjects rules outlined above by Barrett and Carter, for it to proceed anyway it would need to do so on the understanding (1) that any predictable harm be decisively outweighed by social gains; (2) that subjects be fully informed of the risks; and (3) that compensation be paid to cover any damages incurred. Necessarily, this all requires careful consideration, strong oversight from review boards (including in the country where the research will take place), and deep participant engagement to ensure that the project really will be beneficial and is able to minimise risk.

With unintended consequences, it is necessarily the case that we can never have full knowledge about what may harm or cause distress to others, not least because unexpected circumstances may arise ( Iphofen, 2011: 23). However, a researcher can familiarise themselves with the context in which their research will take place and conduct a full, informed and participatory risk assessment, asking all the questions outlined above and many more. They can also put in place mitigation strategies and a risk management plan that are continually updated and which serve as clear guides for project implementation ( Iphofen, 2011). This should include analysis of the potential misuse of research results and assurances that such a risk is low. Likewise, researchers can develop unexpected findings policies and put in place ethical governance structures that support and oversee project implementation.

### Side-effects of randomisation

What does the literature say about how to deal with the effects of randomisation? And how might a pilot build on the literature’s recommendations? On the first question, the literature is reasonably clear – avoid RCTs if you can, for scientific as well as ethical reasons. Deaton is not alone in attributing “no special ability [to RCTs] to produce more credible knowledge than other methods” ( Deaton, 2009: 1), while Barrett & Carter (2010: 524) speak for many when they question the internal validity of RCTs on the grounds that human agency makes the measurement of treatment against effect significantly more challenging than in the biomedical sciences. Many alternative approaches are recommended, of which one of the more promising is contribution analysis (CA).

CA differs from RCTs in that it does not seek a counterfactual explanation of causality (establishing what would have happened had the intervention not taken place), but rather builds a ‘contribution story’ about how an intervention contributes (or not) to change – in other words, whether and how it works, for whom, and under what circumstances ( Ton, 2017: 121). CA was developed by John Mayne in response to the limitations with and frequent inappropriateness of experimental design ( Mayne, 2011; Mayne, 2012; Mayne, 2015). It follows the seven steps outlined Table 1 and is best understood as an overarching framework to guide the use of any preferred combination of individual methods.

**Table 1.** Steps in contribution analysis (Table adapted from method and diagram outlined in Ton, 2017).

Step 1	Set out the specific cause-effect questions to be addressed.
Step 2	Develop robust theories of change for the intervention and its pathways.
Step 3	Gather the existing evidence on the components of the theory of change model of causality.
Step 4	Assemble and assess the resulting contribution claim, and the challenges to it, including alternative theories.
Step 5	Seek out additional evidence to strengthen or challenge the contribution claim.
Step 6	Revise and strengthen the contribution claim.
Step 7	Return to Step 4 if necessary.

If one is committed to using an RCT, however, the literature is explicit that doing so must, as mentioned above, involve “negligible consequences [for participants], unambiguous scientific need for the study and its experimental design, and particular importance of the results” ( Baele, 2013: 24). Following established practice in medical research, some also argue that assessment of the second of these criteria should revolve around equipoise, which means that researchers are genuinely uncertain as to the expected impact and benefit deriving from the intervention(s) ( Abramowicz & Szarfarz, 2020) and must arrive at this conclusion “after having reviewed the available research in the field” ( McKenzie, 2013: para 5)’. Alternatively, they must offer control groups compensation that equals what was gained by the treatment ^7^.

### Instrumentalisation of participants

According to Baele, instrumentalisation is “a fundamental ethical issue…a moral wrong” [that involves] using people as means towards an end” ( Baele, 2013: 25–26). As discussed above, a participant can be conceived of as being instrumentalised in a study when the study has nothing to do with their lives and offers them no benefit. By contrast, if the study benefits participants “such that they are not mere pawns in a trial that will have no bearing on their own realities” ( Baele, 2013: 27), and if they have offered their fully informed consent for participation, then one may consider the study legitimate in that it treats participants with respect and as partners in the research.

Numerous scholarly traditions have reflected on how researchers can go about doing this, ensuring fairness, benefit sharing, reciprocity or justice in what they do, including feminism (e.g., Adkins, 2004), anthropology ( Scheper-Hughes, 1995), education (e.g., Hale, 2008), action research (e.g., Burns, 2012), and post-coloniality (e.g., Tuhiwai Smith, 1999). A key point of reference that draws on each of these traditions is the Global Code for Research in Resource Poor Settings ( https://www.globalcodeofconduct.org/) ( Schroeder *et al.,* 2019). Aiming to end the practice of ‘ethics dumping’, the global code provides guidelines for conducting research with fairness, respect, honesty, and care. This places emphasis on the local relevance of research, co-ownership of that research, clear pathways for feedback around findings, formal knowledge transfer agreements, and established benefit sharing mechanisms.

### Informed consent

Although informed consent is widely considered the *sine qua non* of ethical research, plenty of projects fail to obtain it. This is clearly problematic. Hewlett defines consent as the “autonomous authorisation by one person to permit another person to carry out an agreed procedure which affects the subject” ( Hewlett, 1996: 232). Following this, she considers consent to be ‘genuine and therefore ethically acceptable’ only when four conditions pertain. These are:

1.   The subject has to be mentally, intellectually, and emotionally competent to understand the full scope of the experiment.

2.   Sufficient and unbiased information has to be provided to the subject; consent has to be fully informed.

3.   The subject’s understanding of this information has to be perfect, which means that the researcher has to formally assess this understanding in some way or another.

4.   Participation has to be unambiguously voluntary; this is stressed because participants are sometimes so vulnerable that consent is not genuine.

Humphreys agrees with this position, citing formal US research rules which view consent as rooted in “information, comprehension and voluntariness” and an integral component of “respect for persons” ( Humphreys, 2015: 100). In his guidelines for the European Commission, Iphofen concurs, also noting the importance of subjects choosing ‘freely’, based on “sufficient mental capacity to make such a judgement”, adequate “information about the research” and “that they can understand what that information implies for their involvement” ( Iphofen, 2011: 29).

However, although there is agreement over what consent involves and the fact that it is important, ^8^ there is less agreement over how it should be obtained. Formal ethical guidelines typically expect written consent and consider written agreements as a kind of gold standard. But, as Iphofen observes, there are myriad real-world scenarios where written consent is neither possible nor appropriate:

“Formality could alienate some potential participants who might fear the researcher is a representative of “officialdom” and who might be wary of such engagements. Indeed, some anthropologists complain that they are aware that asking for a signature would be seen as offensive in the communities they study” ( Iphofen, 2011: 29).

This is undoubtedly accurate, and the researcher has to balance the obligation to demonstrate to review boards that consent has been obtained and care for participants who may find traditional consent-gathering mechanisms threatening. One way of doing this is to ensure that the process of seeking and gathering informed consent is witnessed by a third person, and for testimony of this witnessing to be an acceptable verification for review boards. Another is to use a voice recorder. With this, the researcher explains the research, its risks and potential benefits to all participants in terms intelligible to them. The researcher then asks participants to state their name, the date, and the consent they have offered into a voice recorder, with the explanation that this recording will be securely stored solely for the purpose of ‘proving’ that consent was offered.

Whichever method one uses, the anthropological literature is agreed that consent should be seen as a process, not an event ( Iphofen, 2011: 29). This is especially important, as Boyden & Ennew (1997: 41) argue, with children and others in socially subordinate positions (common to participants in cash transfer trials), since they are often less able to exercise or indeed recognise their right to refuse to participate. This entails checking with participants repeatedly during the research encounter to make sure that they feel comfortable continuing and offering them the chance to stop at any point if so desired ( McCormick, 2012).

### Researcher accountability

Another issue for reflection here is that of researcher accountability. As discussed above, it is widely acknowledged that foreign researchers may abuse their power and privilege to act in ways that they would not in their home countries (e.g., Mosse, 2013). This certainly includes those involved in experimental social science research. To ensure researcher accountability, Article 10 of the reference-point Global Code for Research in Resource Poor Settings states that “local ethics review should be sought wherever possible” ( Schroeder *et al.,* 2019), irrespective of whether ethics approval has already been gained in the lead researcher’s high-income home country. Likewise, Articles 12 and 19 remind us that respectful, effective informed consent and risk management procedures are essential. Beyond these basics, Article 13 states that:

“A clear procedure for feedback, complaints or allegations of misconduct must be offered that gives genuine and appropriate access to all research participants and local partners to express any concerns they may have with the research process. This procedure must be agreed with local partners at the outset of the research” ( Schroeder *et al.,* 2019).

In cash transfer trials and related research, this would entail establishing clear understanding between partners of their roles and responsibilities, the clear articulation to participants of their right to report concerns, and the mechanisms by which they can do so, and monitoring to ensure that such mechanisms function. Finally, given that unexpected harms may occur, it is also essential for projects to have in place clear and effective pathways of redress, including insurance policies that compensate in such cases ( Baele, 2013: 27–8).

### Decolonising methodologies to inform design and implementation

The final issue for discussion here regards coloniality and attempts to mitigate for and move beyond it, as per contemporary efforts towards ‘decolonisation’ ( Connell, 2017). In their online essay, Hammond notes that “Decolonisation itself refers to the undoing of colonial rule over subordinate countries” ( Hammond, 2018). However, decolonisation also has a wider meaning beyond ‘the “freeing of minds from colonial ideology”’, such that it has become “a powerful metaphor for those wanting to critique positions of power and dominant culture” ( Hammond, 2018). This translates into the reflexive questioning of received ideas, an interrogation of the standpoint from which contemporary and historical discourses are constructed, the search for alternative epistemologies and ontologies, and the striving for more democratic, inclusive, participatory forms of knowledge generation in the service of emancipation ( Tuhiwai Smith, 1999). This includes approaching the research endeavour in an energy of true partnership, with respect for all participants, and an intention to benefit and include the voices of particularly the most vulnerable or marginalised. One way to attempt this in a cash transfer trial may be to support such communities to reverse the standpoint of analysis of their circumstances, co-theorising with them and, in collaboration with them, taking their theory ‘upwards’ to political actors, using the very methodology that scholars such as Zavala and Tuhiwai Smith praise as decolonising – community-centred participatory action research (PAR).

As Zavala notes, “PAR is part of the broader legacy of activist scholarship and action-research, and can be traced to anti-colonial movements” ( Zavala, 2013: 57). Implicit within it is “the potential for transforming not just the process of knowledge production and the hierarchical relations that exist between university and community, between researchers and researched, but an expansion of the goals of traditional social research” ( Zavala, 2013: 59). In Tuhiwai Smith’s terms, this entails “a collaborative approach to inquiry or investigation that provides people with the means to take systematic action to resolve specific problems” ( Tuhiwai Smith, 1999: 127). In other words, it is an approach to research which is action-oriented, open-ended, co-operative, respectful, and committed to reciprocity ( Burns, 2012; Keane *et al.,* 2017). This has methodological implications that push researchers in considerably more qualitative and collaborative directions than is often the norm in trials and their evaluation.

## Conclusion

### Can the risks ever be justifiable?

This paper has sought to articulate the many ethical questions facing cash transfer piloters. It further asks whether such pilots can ever be ethical and if so how. The literature reviewed strongly suggest that the answer will depend on context. The risks to potential pilot participants are of paramount importance and must be weighed against the potential benefits to them and to society. In addition, it is essential that pilots respect standard human subjects rules. As Barrett and Carter explain, these rules state: “(1) that any predictable harm be decisively outweighed by social gains; (2) that subjects be fully informed of the risks; and (3) that compensation be paid to cover any damages incurred” ( Barrett & Carter, 2010: 520). The above review suggests that this will involve significant, structured attempts to avoid and mitigate harm, which begin with rigorous, respectful informed consent processes and may include avoiding RCTs, targeting and conditionality entirely. In addition, researchers and pilot designers will need to pay attention to power relations and build genuine forms of reciprocity and partnership into what they do, of the kind advocated by the Global Code for Research in Resource Poor Settings.

Should these rules be respected, a case could likely be made for a given pilot being ethical. That being said, it is worth heeding the words of Gokah (2006) and Iphofen, who argue that “the only realistic way for researchers to conduct an assessment of this [ethical] balance is to adopt a continual reflexive stance in order to conduct an ongoing estimate of harms and benefits and make both research and personal action judgements accordingly” ( Iphofen, 2011: 26). Research, including experimental research around cash transfers, is a dynamic, living process and a commitment to fairness, respect, care, and honesty requires that the researcher continually reflect on what is happening and how, with an openness either to changing course or to stopping entirely if necessary. This openness must be a central commitment for those pursuing cash transfer trials.

## Data availability

No data are associated with this article.

## Footnotes

^1^ This can be understood as research which seeks ‘to actively experiment, in real-life situations, theoretical hypotheses in order to test their validity and produce more useful knowledge (than that provided by non-experimental research) for policy-makers of all sorts (governments, NGOs, philanthropists, international organisations, etc.)’ ( Baele, 2013: 3).

^2^ Even if that claim is widely disputed and has arguably been discredited (e.g., Deaton, 2020).

^3^ There is, however, a commonly recycled argument in favour of conditionality that goes beyond the need to allocate limited resources effectively, which can accurately be described as paternalistic. This is the argument that, without appropriate ‘guidance’, transfer recipients will waste their newly acquired money on damaging temptation goods such as cigarettes and alcohol, causing harm to themselves and to others. Following this, conditionalities are defended as an ethical, protective necessity. Yet despite the wide reach of this argument, it has in fact been comprehensively disproved by empirical research on all continents. It should thus be discounted (see Evans & Popova, 2017 for a meta-study on the question; see also Davala *et al.,* 2015 for a detailed case study).

^4^ There are numerous infamous examples of experimental RCT projects giving financial inducement for behaviour that is either illegal or socially damaging, e.g. Bertrand *et al.,* 2007 some of which are cited in Ravallion (2014) and Özler (2014). Humphreys (2015) also covers a handful.

^5^ Scholars within the social sciences and humanities have for some time now problematised the notion of knowledge as an abstract form of truth that an abstract form of research can uncover. Rejecting the positivism of much canonical scholarship, those influenced by the linguistic turn have come to understand knowledge discursively – as both artefact and ongoing construction of socio-cultural practice and thus embodying and reproducing relations of power. Although Foucault (1980) is the most frequently cited proponent of this position, it is common to researchers within feminist ( Aradau, 2004; Aradau, 2008), anthropological ( Howard, 2016), post-structural ( Howarth, 2013), critical race ( Mills, 1998) and indigenous ( Tuhiwai Smith, 1999) traditions.

^6^ There is much to recommend this approach, although it too is subject to considerable academic critique (e.g., Olivier de Sardan & Hamani, 2018).

^7^ However, there is dissent within the literature over this – Fries & Krishnan (2004), for example, reject equipoise and argue that genuine informed consent is what makes experimental research unproblematic, while Miller & Brody (2003) suggest that ethical determination should depend primarily on an assessment of risks.

^8^ It is also accepted that, under certain special and very well-justified circumstances, the requirement for consent can legitimately be relaxed – for example, research on illicit activities which would be impossible if the researcher were open about their aims. These exceptions do not concern the present discussion and so are omitted (see Iphofen, 2011 for a fuller reflection on these matters).
