# Supplementary material for: Towards ethical good practice in cash transfer trials and their evaluation
Source: Open Res Eur. 2022 Mar 21;2:12. Originally published 2022 Jan 25. [Version 2] doi: 10.12688/openreseurope.14258.2 (PMC10446089; doi:10.12688/openreseurope.14258.2)
Supplement: Peer review history

# Peer review: Reviewer response for version 1

**DOI:** 10.21956/openreseurope.15379.r28454

**Published:** 2022-02-28

**Authors:** Maria Elisa Balen

**Affiliations:** 1 Universidad Nacional de Colombia, Bogotá, Colombia

Neil Howard’s reflection about ethical good practice in cash transfer trials and their evaluation is much welcome for it both highlights an important issue of concern and brings together diverse discussions regarding these programs. Not only does he specify some of the challenges researchers face in the field, but points to the ways in which they may be overcome -which are not necessarily straightforward, or reducible to ticking a box. Bringing together insights from both RCTs in general and trials and evaluations of cash transfers in particular, the article locates the ethical discussions in specific issues related to cash transfers, which permits both exemplification and the elaboration of grounded reflection: these properties make the article both a good introduction to new practitioners and a worthwhile read for those of us who have been engaged in this field for some time now.

 When one considers reflections about ethics and cash transfer programs in this broad way, it becomes necessary to locate particular interventions within wider practices in which they are inscribed -or which have been seen on various occasions to piggy-back on these interventions. In particular, and given that part of the success of CCTs is predicated on their cost-effectiveness, it is worth considering what this ‘cheapness’ can imply. What various ethnographic studies compilated in our ‘Money form government in Latin America: conditional cash transfers and rural lives’ (2021) note, on the one hand, is how a particular cash transfer can be inscribed not only in past experiences but in expectations about future cash transfers: participants can anticipate that the outcome of their engagement in one intervention can have consequences for future ones, in terms of for example being on the list of potential targeting or not, and how they appear on that list. Understanding the consequences of an intervention requires, thus, broadening the scope in time. On the other hand, in contexts where local functionaries are pressed to meet institutional metrics yet count with scarce resources to do so, they have been known to ‘add’ conditionalities to the CCTs. An example of this, as Tara Cookson notes in the above-mentioned book, is pregnant women in rural Peru having to walk considerable distances to arrive at clinics in order to give birth -clinics which are often closed- in order to keep the subsidy and so local functionaries can tick an extra metric. If you take both the wider implementation issue and the historical dynamic, then addressing cash transfers as a form of government comes into view.

 Whilst it can be problematic to tie research ethics to specific theoretical approaches, the last section on decolonizing methodologies and the work of Zavala and Smith can be read as an invitation to move away from considering CCT implementation and evaluation as technical interventions and considering not only the programs but associated researchers as part of the circulation of money and information these imply, and which is not only traversed by power asymmetries but generative of relations, practices and dynamics that are worth investigating and reflecting upon.

Are all factual statements correct and adequately supported by citations?

Yes

Is the review written in accessible language?

Yes

Are the conclusions drawn appropriate in the context of the current research literature?

Yes

Is the topic of the review discussed comprehensively in the context of the current literature?

Yes

Reviewer Expertise:

Sociology of Money and Debt

I confirm that I have read this submission and believe that I have an appropriate level of expertise to confirm that it is of an acceptable scientific standard.

## References

[1] : Money from the Government in Latin America Conditional Cash Transfer Programs and Rural Lives. *Routledge* .2021;

# Peer review: Reviewer response for version 1

**DOI:** 10.21956/openreseurope.15379.r28470

**Published:** 2022-02-17

**Authors:** Karl Widerquist

**Affiliations:** 1 Georgetown University-Qatar, Doha, Qatar

This is an excellent paper. It makes an important point about social science experiments—a point that has been largely ignored in the literature. For example, I wrote an entire book on UBI experiments, and I didn’t even think of mentioning this issue. If it wasn’t for the typos and grammatical errors, it could be disseminated as is. I will not point out all the many grammatical issues that need to be fixed, but I’ll point out *two* just in *the last paragraph* as an example of the kind of proofreading work that needs to be done to get this excellent paper ready for wider dissemination. I will also suggest one substantive change that isn’t essential but might improve the paper.

 Grammatical issue 1: The following sentence has two verbs in one clause (is and requires). I think the author needs to make it two clauses or two sentences: “Research, including experimental research around cash transfers, is a dynamic, living process and a commitment to fairness, respect, care, and honesty requires that the researcher continually reflect on what is happening and how, with an openness either to changing course or to stopping entirely if necessary.”

 Grammatical issue 2: This sentence has no verb at all. I think the author wants the word “be” in there somewhere: “This openness must a central commitment for those pursuing cash transfer trials.”

 Here’s the substantive issue. The reason I didn’t mention this issue in my book critically discussing UBI trials is that I assumed this kind of experiment can’t significantly harm people: the experiment group gets money, which is an unequivocal good and the control group gains and loses nothing. I suppose the control group could become jealous of the experimental group—but there are lots of way better-off people that low-income people can be jealous of and those groups are a lot better off than the experimental group. So, I’m thinking: no harm, no foul. Although the paper is strong now, I think the article would be stronger if it explained how someone might feel the way I have (over the last 20 years of reading about UBI trials) and then explained to people like me how participants in UBI trials might be harmed even though it seems like the worst possible outcome is no worse than being uninvolved in a trial.

Are all factual statements correct and adequately supported by citations?

Yes

Is the review written in accessible language?

Yes

Are the conclusions drawn appropriate in the context of the current research literature?

Yes

Is the topic of the review discussed comprehensively in the context of the current literature?

Yes

Reviewer Expertise:

Distributive justice, Universal Basic Income (UBI), and UBI experiments. I’m much better at assessing UBI trials than other social science experiments

I confirm that I have read this submission and believe that I have an appropriate level of expertise to confirm that it is of an acceptable scientific standard.
